# De novo genome assembly and genome skims reveal LTRs dominate the genome of a limestone endemic Mountainsnail (*Oreohelix idahoensis*)

**DOI:** 10.1186/s12864-022-09000-x

**Published:** 2022-12-02

**Authors:** T. Mason Linscott, Andrea González-González, Takahiro Hirano, Christine E. Parent

**Affiliations:** 1 Department of Biological Sciences, University of Idaho, Moscow, ID USA; 2 Institute for Interdisciplinary Data Sciences, University of Idaho, Moscow, ID USA; 3 Department of Biology, University of Florida, Gainesville, Florida USA; 4 Center for Northeast Asian Studies, Tohoku University, Sendai, Miyagi Japan

**Keywords:** LTR expansion, *Oreohelix*, Comparative genomics, Genome skim, Gene family evolution, Limestone

## Abstract

**Background:**

Calcareous outcrops, rocky areas composed of calcium carbonate (CaCO_3_), often host a diverse, specialized, and threatened biomineralizing fauna. Despite the repeated evolution of physiological and morphological adaptations to colonize these mineral rich substrates, there is a lack of genomic resources for calcareous rock endemic species. This has hampered our ability to understand the genomic mechanisms underlying calcareous rock specialization and manage these threatened species.

**Results:**

Here, we present a new draft genome assembly of the threatened limestone endemic land snail *Oreohelix idahoensis* and genome skim data for two other *Oreohelix* species. The *O. idahoensis* genome assembly (scaffold N50: 404.19 kb; 86.6% BUSCO genes) is the largest (~ 5.4 Gb) and most repetitive mollusc genome assembled to date (85.74% assembly size). The repetitive landscape was unusually dominated by an expansion of long terminal repeat (LTR) transposable elements (57.73% assembly size) which have shaped the evolution genome size, gene composition through retrotransposition of host genes, and ectopic recombination. Genome skims revealed repeat content is more than 2–3 fold higher in limestone endemic *O. idahoensis* compared to non-calcareous *Oreohelix* species. Gene family size analysis revealed stress and biomineralization genes have expanded significantly in the *O. idahoensis* genome*.*

**Conclusions:**

Hundreds of threatened land snail species are endemic to calcareous rock regions but there are very few genomic resources available to guide their conservation or determine the genomic architecture underlying CaCO_3_ resource specialization. Our study provides one of the first high quality draft genomes of a calcareous rock endemic land snail which will serve as a foundation for the conservation genomics of this threatened species and for other groups. The high proportion and activity of LTRs in the *O. idahoensis* genome is unprecedented in molluscan genomics and sheds new light how transposable element content can vary across molluscs. The genomic resources reported here will enable further studies of the genomic mechanisms underlying calcareous rock specialization and the evolution of transposable element content across molluscs.

**Supplementary Information:**

The online version contains supplementary material available at 10.1186/s12864-022-09000-x.

## Background

Calcareous rocks such as limestone, marble, or dolomite are required ingredients for cement production and thus are necessary ‘building blocks’ for infrastructure. Regions containing these valuable resources often host a diverse and specialized group of species that have adapted to the unique abiotic conditions present in calcareous habitats [1, 2]. A product of this specialization is that many calcareous rock endemics are characterized by narrow ranges (e.g. a single hill, cave, or outcrop) which increases the risk of extinction from disturbance [3]. Balancing the societal needs for carbonate rock and habitat requirements of calcareous rock endemic species can be particularly challenging as there may be little middle ground between economic interests and biodiversity preservation. While there is growing global interest to safeguard calcareous rock endemic diversity [4], government and private quarrying has already caused the extinction of more than 20 calcareous rock endemic species with more likely to follow [5, 6].

Central to this industry-biodiversity conflict is determining what level of conservation priority should be given to species that are edaphically specialized to calcareous habitats. While many calcareous rock endemics are clearly threatened, resources available for conservation actions are limited. Calcareous edaphic specialist species are often, but not always, members of recent radiations that are spread across several calcareous outcrops [2]. A large number of closely related edaphically specialized species spread across numerous isolated outcrops can make targeted species conservation infeasible with the resources and political will available [7]. Instead, policymakers may opt to preserve the most species rich outcrops and allow development to proceed on less diverse areas [5, 8]. However, this conservation approach omits considerations of the evolutionary processes generating calcareous rock endemic diversity. The process of edaphic speciation can be abrupt (i.e. polyploidy, chromosomal rearrangement or inversions) or relatively gradual (i.e. polygenic loci under divergent selection over several hundred generations) (reviewed in [2, 9, 10]). Failure to account for these evolutionary processes in conservation plans of calcareous rock endemics may result in misapplying protective actions to morphologically distinct ecotypes of existing species and/or permitting the destruction of habitat for fully reproductively isolated edaphically specialized species [2].

One of the major groups of calcareous rock endemics that are increasingly being threatened by development are land snails [8, 11]. More than a quarter of all IUCN Red List land snail species with near threatened or higher threat assessments appear to reside primarily or exclusively on calcareous substrates (371 of 1460 species) [12]. Unlike other edaphic specialists that must physiologically and morphologically adapt to tolerate higher concentrations of calcium carbonate (CaCO_3_) in calcareous habitats, land snails must acquire sufficient CaCO_3_ from the environment to biomineralize their shell [13] and may exploit greater environmental availability of CaCO_3_ at calcareous outcrops to increase biomineralization output [14]. Indeed, a number of calcareous rock endemic land snails express thickened or elaborate calcareous ornaments (e.g. prominent calcareous ribs or keels) [15] which may require an abundance of environmental CaCO_3_ to develop normally. Edaphic specialization by land snails to calcareous habitats may be distinct from classical examples of calcareous edaphic evolution in that land snails are often specializing to an environment favorable to their physiological needs and not to one requiring substantial metabolic adaptations [2]. Given these differences, the genomic mechanisms underlying calcareous edaphic specialization in land snails may be broadly dissimilar from those identified in genomic studies of other calcareous edaphic specialists. Key to unravelling the genomic mechanisms associated with edaphic specialization are genomic assemblies which provide important context for evolutionary and functional inference. Unfortunately, the scarcity of genomic resources available for calcareous rock endemic land snails has hindered our understanding of the origins of edaphic diversity.

An ideal system to study the process of calcareous edaphic specialization in land snails should have repeated transitions to calcareous rock environments to serve as replicates and transitions at different stages of the edaphic specialization process. *Oreohelix* ‘Mountainsnails’ are suitable candidates as there are numerous transitions from a smooth form which occurs on a variety of geologic substrates to various ornamented forms that predominantly inhabit calcareous substrates [16], have hybrid zones at calcareous outcrop boundaries between forms that indicate recent divergence [17, 18], and phylogenetic studies indicate some ornamented species are of considerable age [16]. Furthermore, many highly ornamented species are declining in population size [18] and are considered to be highly threatened by conservation authorities [19]. Uncovering the processes involved in edaphic specialization in *Oreohelix* would have considerable bearing on the systematics and conservation status of its members.

Here, we assembled a draft genome assembly of the costate Mountainsnail, *Oreohelix idahoensis*, a threatened limestone endemic land snail species from the Northwest United States that expresses thickened shell ornaments. In comparison to other gastropod genome assemblies, there were several significantly expanded gene families putatively related to stress response and biomineralization. We also show from analyses of *Oreohelix* genomes skims and comparisons to other mollusc genome assemblies that repeat content is substantially elevated in the *O. idahoensis* genome. The genomic resources reported here will serve as a foundation for the conservation genomics of this threatened species and for understanding the genomic basis of resource constrained biomineralization.

## Results

### Genome assembly and annotation of *O. idahoensis*

Through utilizing PacBio CLR long reads (324.5 Gb) and 10X genomics linked reads (425.8 Gb), we generated a relatively contiguous and complete genome assembly of *O. idahoensis* (Table 1). Genomescope 2.0 [20] analysis of 31-mer counts estimated that the *O. idahoensis* genome was 7.01Gb in size, has a heterozygosity of 0.51% (Supplementary Fig. 1), and a repeat content of 74.9%. Estimates of genome size using raw read mapping rates using ModEst however showed the genome size to be 8.49 Gb [21]. The final pre-polished assembly was produced by merging the hybrid Supernova-DBG2OLC [22] assembly produced from both long and linked reads with the canu v.1.9 [23] assembly generated solely from long reads (Supplementary Table 1). The subsequently polished genome draft was 5.40 Gb in size and was composed of 23,228 scaffolds (scaffold N50 of 404.19 kb). The high repeat content of the genome draft is a likely contributing factor for the difference between the final assembly size (5.40 Gb), the estimated genome size using k-mers (7.01 Gb), and estimated genome size using back mapping rates (8.49 Gb) as repetitive regions are prone to collapse during assembly [23]. The estimated heterozygosity of the *O. idahoensis* draft assembly using genomescope was lower than other published land snail genomes (e.g. *C. nemoralis*: 1.42%; *C. unifasciata*: 1.09%) [24, 25], which likely reflects the isolation and small population size of this threatened species [26]. The draft genome was fairly complete with regard to coding regions as 86.6% of metazoan Benchmarking Universal Single-Copy Orthologs (BUSCO) genes were single-copy or duplicated in the assembly (single copy: 77.3%, duplicated: 9.1%, fragmented: 6.6%) [27]. In terms of contiguity and BUSCO scores, the *O. idahoensis* genome draft assembly compares favorably to many other large molluscan genomes (Table 1).

**Table 1:** Genome assembly statistics of the *O. idahoensis* genome draft and closely related or large molluscan genomes

Genome assembly	Size (Gb)	Scaffold N50 (Kb)	BUSCO score	Source
*Oreohelix idahoensis*	5.40	404	86.6	This study
*Euprymna scolopes*	5.11	3724	96.9	Belcaid et al. 2019
*Callistoctopus minor*	5.09	466	76.2	Kim et al. 2018
*Cepaea nemoralis*	3.49	330	87.2	Saenko et al. 2021
*Candidula fasciata*	1.29	246	92	Chueca et al. 2021

The final annotation set produced by integrating transcript, protein and ab-initio evidence contained 27,692 predicted protein-coding genes which is within the range of other known gastropod genome assemblies [25, 28]. Mean gene size was 34,594 bp which is roughly three times larger than previous land snail genome assemblies (e.g. *C. nemoralis* mean gene size: 9629 bp; *C. unifasciata* median gene size: 11,931 bp) [24, 25], reflecting the influence of repetitive elements on intron size (78.44% of genes had repetitive elements nested in genic regions; Table 2). A total of 92.1% of all genes had a hit to either the NCBI non-redundant nucleotide database, InterProscan [29], or UniProt (Supplementary Table 2). Scanning of scaffolds for contamination with conterminator v.1.0 [30] revealed a single potential 1.2 kb insert of *Escheria coli* DNA in one scaffold of the assembly. Blasting of the contaminated region against the NCBI nucleotide database did not result in any alignments to *E. coli* sequences so the putative contaminated region was retained in the assembly.

**Table 2:** Gene characteristics of the *O. idahoensis* draft assembly

Feature	Value
Number of protein codeing genes	27,692
Number of mRNAs	46,907
Mean isoforms per gene	1.69
Mean exon number per mRNA	5.6
Mean gene length (bp)	34,594
Mean mRNA length (bp)	31,244
Mean mRNA exon length (bp)	265
Mean intron length (bp)	5523
Number of genes with repetitive element overlap	21,722

### Transposable element content and evolution

**Fig. 1:**
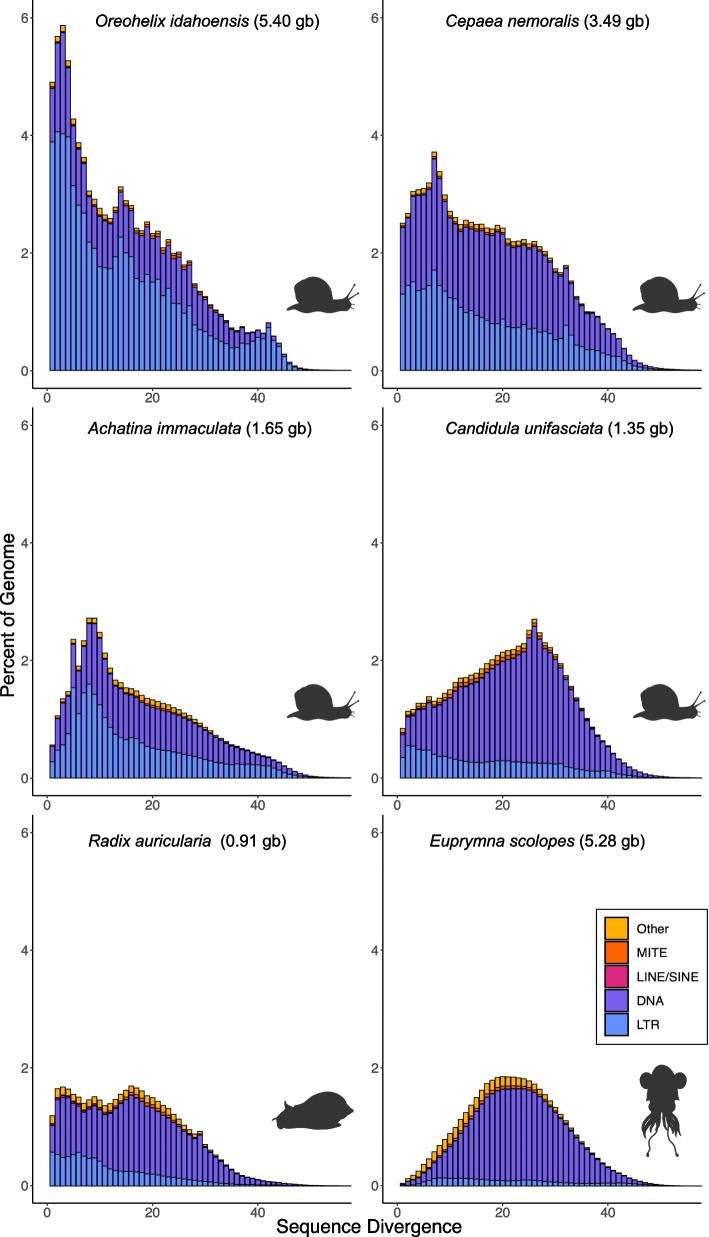
Repetitive element landscapes of *O. idahoensis* and other large or closely related molluscan genomes. Repeat classes on the bottom right. The bottom axis depicts Kimura-divergence from consensus with greater divergence being indicative of past repeat expansion. The left axis measures the total proportion of the genome draft occupied by the repeat class. Outline drawings were created with BioRender

To evaluate the repeat content of the *O. idahoensis* genome draft and place the proportion of identified elements in context with other mollusc genome assemblies, we created custom repeat libraries for *O. idahoensis* and five other previously published mollusc genomes (*Cepaea nemorals, Candidula faciata, Achatina immaculata, Radix auricularia*, and *Euprymna scolopes*) using the EDTA pipeline v.1.9.9 [31]. Repeat content of the *O. idahoensis* genome draft was estimated to be 85.74% which is higher than all currently available mollusc genome assemblies, including those larger than 2.5 Gb [32]. Most of the repeat content in the *O. idahoensis* draft assembly is classified as transposable elements and a small proportion are predicted to be simple and low-complexity repeats (Supplementary Table 3). The most common repetitive elements were long terminal repeat transposons (LTR) which make up 57.73% of the assembly (Fig. 1; Supplementary Table 3) and are predominantly of the Gypsy/DIRS1 family (32.53% assembly size). This estimate of LTR content is well outside of the known range of LTR content across molluscan genomes (previous estimates are 2–8% LTR content) [33]. DNA transposons compose the remainder of the repeat landscape and are largely terminal invert repeat sequences (Supplementary Table 3).

Our reannotations of five other molluscan genomes revealed significantly greater LTR content than was previously reported in each assembly (Fig. 1). This may be a result of previous studies not utilizing a dedicated LTR detection step in their repeat annotation pipeline, which is now recommended or standard practice for several popular repeat annotation programs including RepeatModeler 2 [34] and EDTA. The depauperate number of long and short interspersed nuclear elements (LINE/SINE) in our repeat annotations of prior assemblies compared to previous estimates may be a product of this difference as LINE and SINE elements can be nested within larger LTR elements [35].

We examined repeat expansion and contraction across the six mollusc genome assemblies by comparing the Kimura sequence divergence of each identified transposable element to its consensus element within the repeat library. The repeat landscape of *O. idahoensis* indicates that repetitive elements, predominantly LTRs, have undergone expansion recently (Fig. 1). Using a Kimura divergence cut-off of 5%, over 25% of the *O. idahoensis* genome draft assembly is attributable to recent repeat expansion, primarily LTRs. While some land snail genome assemblies such as *C. nemoralis* and *A. immaculata* showed evidence of repeat expansions of DNA and LTR transposons, neither were to the same degree as *O. idahoensis*. All other mollusc genomes analyzed had older repeat expansions and lower repeat content.

**Fig. 2:**
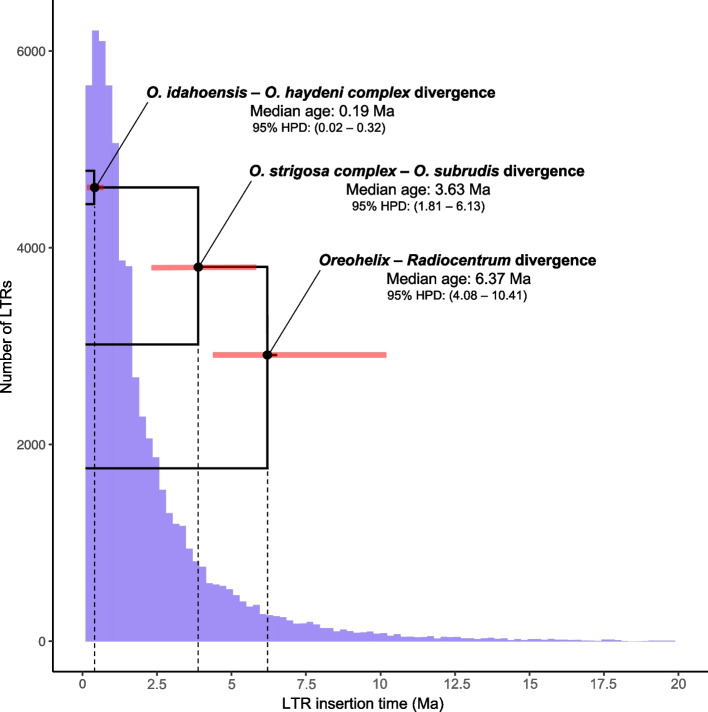
Estimated LTR insertion times in the *O. idahoensis* genome draft and divergence times between major *Oreohelix* groups. Upper and lower 95% highest posterior density divergence are depicted at each node in red. Divergence time estimates are taken from Linscott et al. [16]

To understand how LTRs may be related to species divergence within *Oreohelix,* we compared repeat content estimated from genome skims, LTR insertion times, and *Oreohelix* species divergence estimates from a previous study [16]*.* Analysis of genome skims using RESPECT v. 1.0.0 [36] revealed *O. idahoensis* contains roughly 2–3 fold of high copy repetitive k-mers per million bases compared to a closely related smooth, non-limestone endemic *O. s. strigosa* and a distantly related smooth, non-limestone species *O. jugalis* (Table 3)*.* We estimated LTR insertion times by comparing divergence between LTR sister pairs using a previously established molluscan substitution rate (see Materials and Methods). Most of the LTR insertions in the *O. idahoensis* genome preceded the split of *O. idahoensis* from the *O. haydeni* complex (Fig. 2). 84.57% of estimated LTRs insertion times in the *O. idahoensis* genome occurred after the median age of the *O. strigosa complex – subrudis* split (Fig. 2).

**Table 3:** Estimates of repetitive k-mer content and genome size from *Oreohelix* genome skims

Sample	Genome Size (Gb)	Coverage	HRCM^**1**^
*O. s. strigosa*	6.18	7.54	497
*O. jugalis*- A	6.53	5.75	997
*O. jugalis*- B	6.11	5.95	811
*O. idahoensis*- A	8.99	8.53	1807
*O. idahoensis*- B	9.11	5.60	1595

^1^HCRM stands for high copy repeats per million and is the average count of the 10 most represented k-mers in a sample of a million base pairs. Higher HCRM values are indicative of greater transposable element content

We then calculated the ratio of solo LTRs to intact LTRs to estimate the frequency of ectopic recombination in the *O. idahoensis* genome draft. The presence of solo LTRs indicates that ectopic recombination between LTR elements has occurred and can be compared against the total proportion of intact LTR elements to estimate LTR removal rates and rate of ectopic recombination [37]. We found a high ratio of solo to intact LTR elements present in the *O. idahoensis* genome across LTR families (median ratio: 6.33, Supplementary Table 4), which indicates that there has been a large number of ectopic recombination events that have reduced the number of active LTR elements and facilitated genomic rearrangement.

### Phylogenetic analysis and gene family expansion

**Fig. 3:**
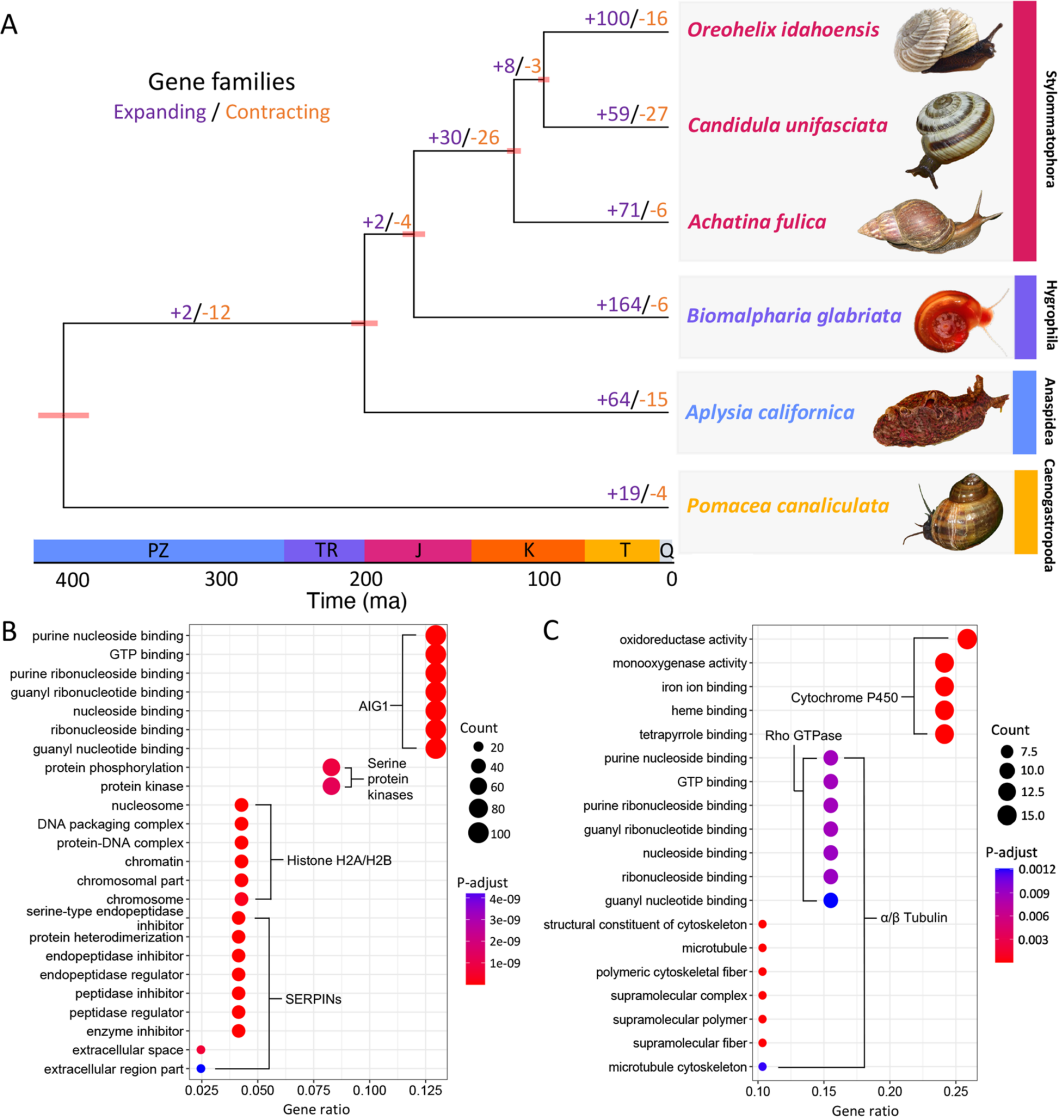
Gene family evolution and GO enrichment analysis of *O. idahoensis* genome draft. **A** BEAST Phylogenetic tree constructed using single-copy orthologs of gastropod species. Upper and lower 95% highest posterior density divergence are depicted at each node in red. Gene family expansion/contraction values are depicted for a given branch with expansion in purple and contraction in orange. Timescale abbreviations: Pz, Permian; Tr, Triassic; J, Jurassic; K, Cretaceous; T, tertiary; Q, Quaternary. **B** GO enrichment analysis of 119 significantly expanded gene families in *O. idahoensis*. **C** GO enrichment analysis of 58 expressed retrocopies with InterProScan GO annotation in the *O. idahoensis* genome. Photo credit: *O. idahoensis,* Richard A. Salsbury; *C. unifasciata,* Sebatsian SANT CC BY-NC 4.0; *A. fulica* Alexander R. Jenner CC BY-SA 3.0; *B. glabriata*, Lewis et al., CC BY 2.5; *A. californica*, Jerry Kirkhart CC BY 2.0; *P. canaliculata*, Cheng Te Hsu CC BY-SA 4.0. Figure layout inspired by [42]

To evaluate the phylogenetic placement of *O. idahoensis*, we first aligned single copy orthologs of six gastropod genomes using Orthofinder v.2.4.0 [38], including two other land snail genomes (*Achatina fulica,* and *Candidula unifasciata,*), a freshwater snail genome (*Biomalpharia glabriata*), a sea-slug genome (*Aplysia californica*), and a Caenogastropod snail (*Pomacea canaliculata*) to serve as an outgroup. Our approach identified a total of 20,598 orthogroups across the six gastropod species, with 1197 single-copy orthogroups across all species and 300 orthogroups specific to *O. idahoensis*. We then constructed an ultrametric time-calibrated phylogenetic tree from the multiple sequence alignment of single copy orthologs produced by Orthofinder using BEAST v.1.8.4 [39] (for further details see Materials and Methods). Overall, our phylogeny of gastropod genomes closely matches those from a previous phylogenetic study in terms of topology and branching events (overlap in 95% highest posterior density estimates of divergence events inferred by [40]; Fig. 3A). Our results show that *O. idahoensis* split from *C. unifasciata* between 82.3 and 93.7 MYA (95% highest posterior density estimate), which is consistent with the earliest fossil evidence of Oreohelicidae in Cretaceous-Tertiary boundary deposits [41].

To better understand gene family evolution within the *O. idahoensis* genome, we used CAFE v.3.03 [43] to analyze gene family expansion and contraction across Gastropoda. We found 115 significant expansions and 39 significant contractions (*P* value < 0.01) on the branch leading to *Oreohelix* (Fig. 3)A). GO enrichment analyses of these expanded orthogroups in clusterProfiler [44] revealed a high proportion of the genes were enriched for GO functions related to nucleotide binding, G-protein coupled peptide receptors, chromatin, and serine-type endopeptidase inhibitors (Fig. 3B). InterProScan identifiers of genes containing significantly enriched GO terms indicated that AIG1, serine protein kinases, histone H2A/H2B, and SERPINs (Fig. 3B) were the primary expanded gene families containing enriched GO terms. The large expansion of AIG1 genes is notable as this gene family is known to rapidly expand and contract across Mollusca and its expression is associated with heat stress and parasitic load [45, 46]. The *O. idahoensis* genome contains 112 partial or complete copies of this gene which is the largest number of AIG1 elements of any molluscan genome [46]. The expansion of histone H2A/H2B genes is also notable as histone variants are often associated with distinct chromatin regions and suggest a diverse toolset for epigenetically regulating transcription [47]. Of final note is the expansion of serine protein kinases and SERPINs, both proteins have been detected in mollusc shell proteomes and mantle tissue across molluscan species [48].

### Retrotransposition of host genes

**Fig. 4:**
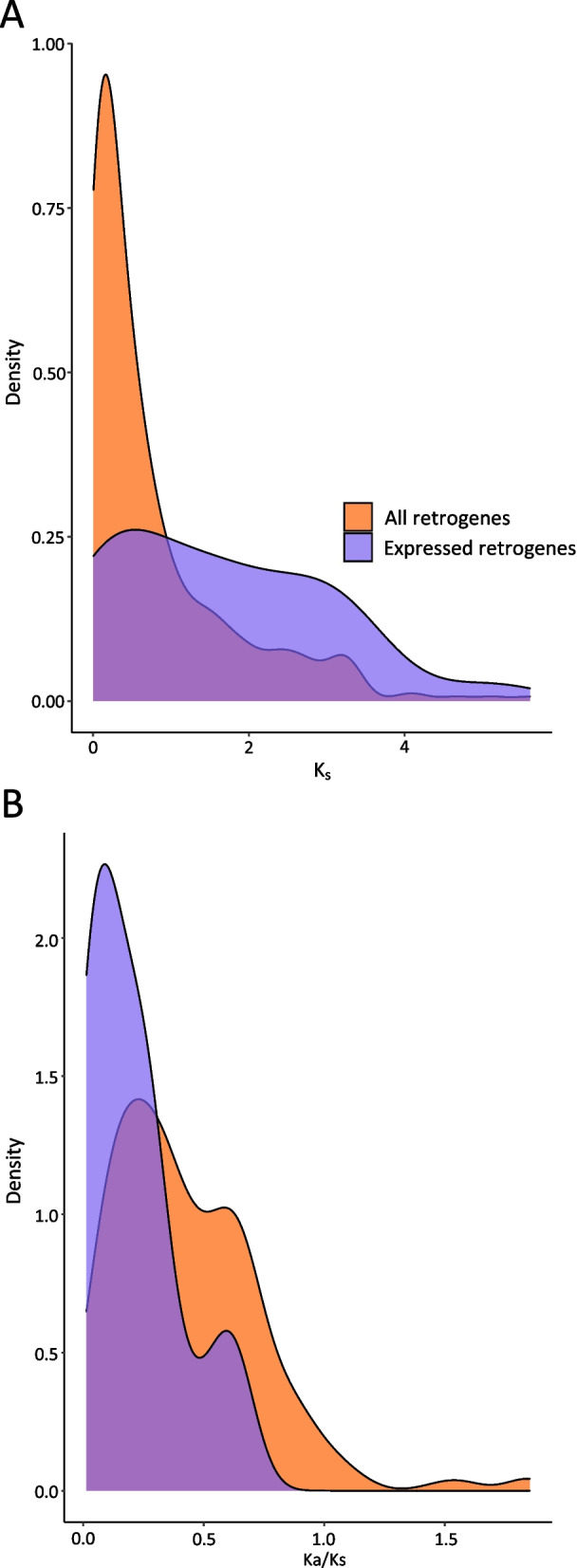
*O. idahoensis* retrocopy Ks divergence and Ka/Ks ratios. **A** Expressed and non-expressed retrocopy kernel-density estimates of Ks divergence. **B** Expressed and non-expressed retrocopy kernel-density estimates of Ka/Ks scores. Ka/Ks values less than 1 indicate purifying selection

Given the large proportion of repetitive elements in the *O. idahoensis* genome draft assembly, we scanned the assembly for retrocopies produced through retrotransposition of host genes using Retroscan v.1.1 [49]. We identified 492 retrocopies of which 22 were intact, 146 were retrogenes, 275 were chimerical retrogenes, and 49 were pseudogenes (see Material and Methods for definitions of retrocopy types). Of these retrocopies, 80 were expressed in the *O. idahoensis* transcriptome (see Materials and Methods). The distribution of synonymous mutations (Ks) across retrocopies inferred using Retroscan revealed most retrocopies are recent and that expressed retrocopies can be of older age (Fig. 4A). The ratio of synonymous to non-synonymous mutations (Ka/Ks) for expressed retrocopies indicate that they appear to largely be under purifying selection as there was no expressed retrocopy with a Ka/Ks ratio greater than 1 (Fig. 4B).

GO enrichment analyses of the expressed retrocopies using ClusterProfiler revealed that monooxygenase activity, GTP binding, and microtubule cytoskeleton GO terms were significantly enriched. These GO terms were associated with InterProScan classified gene families of cytochrome P450, Rho GTPases, and α/β tubulin (Fig. 3C). Many of the GO terms within expressed retrocopies overlapped between genes, likely reflecting the fusion of different genes in chimeric retrocopies [49].

### Genome size and LTR expansion

The *O. idahoensis* genome is estimated to be more than twice the size of other previously published land snail genome assemblies [50]. To determine whether the large size of the *O. idahoensis* genome draft assembly is a product of an ancient whole genome duplication event in the branch leading to *O. idahoensis,* we estimated ancient whole genome duplication using Ks plots of whole paranome sequences and a molluscan nuclear substitution rate in WGD v.1.0.1 [51]. We detected a single peak in our Ks plot (1.1–1.5; Supplementary Fig. 2) which was dated to be approximately 66–91 MYA. This age estimate is consistent with the established ancient whole genome duplication at the base of Styllomatophoran land snails detected by previous studies [40, 52].

We examined the possibility of recent whole-genome duplication using Gaussian Mixture Models of different ploidy models of heterozygous SNPs distributions using nQuire [53]. The best performing ploidy model was a diploid model as it had the lowest delta likelihood from the free model (diploid delta likelihood: 698275; triploid delta likelihood: 78931; tetraploid delta likelihood 1,375,106). These results suggest the large size of the *O. idahoensis* genome is likely not attributable to an ancient whole genome duplication specific to Oreohelicidae or to a recent whole genome duplication event.

Finally, we examined genome size estimates of the closely related *O. s. strigosa* and distantly related *O. jugalis* by analyzing k-mer counts of genome skims using RESPECT v.1.0.0 [36]. Genome size estimates were roughly 1.5 times greater for *O. idahoensis* genome skims compared to either smooth form species (~ 9 Gb: ~ 6 Gb; Table 3). While the specific base pair estimates for genome size from genome skims may be off for all *Oreohelix* due to their relatively high repeat content [36], the relative differences in estimated genome size of *O. idahoensis* and other *Oreohelix* species is likely real given their stark differences in repetitive k-mer content.

### Historical demography of *O. idahoensis*

**Fig. 5:**
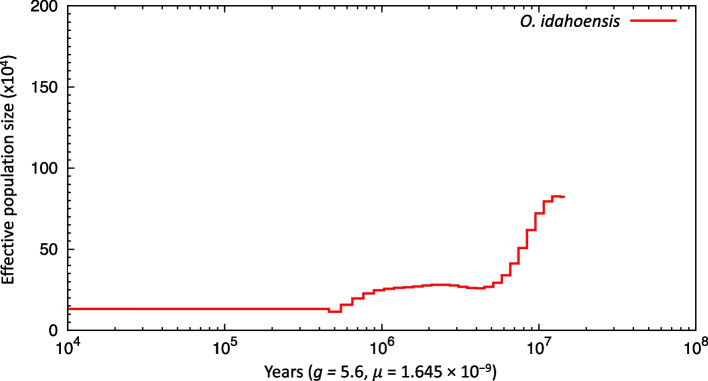
*O. idahoensis* historical effective population size (Ne) trajectory obtained from PSMC

Given the low heterozygosity and threatened status of *O. idahoensis*, it may be expected that this species has undergone a severe population bottleneck recently. Historical demography trends revealed by PSMC [54] revealed substantial decline in effective population size in the last several million years followed by stasis (Fig. 5). Both PSMC and heterozygosity estimates indicate *O. idahoensis* has a low effective population size that may be a product of a population bottleneck and/or increased selfing.

## Discussion

Elucidating the genomic features associated with adaptive divergence can be challenging without suitable genome assemblies to provide evolutionary and functional context. In this study, we generated a high quality draft-assembly of the limestone endemic land snail *O. idahoensis* and genome skims from two other *Oreohelix* species to understand the genomic features underlying edaphic specialization in land snails. Unlike other studies of sessile edaphic specialists (e.g. serpentine or karst plants) [55], in this study we do not find that any evidence of a recent or ancient whole genome duplication event specific to *O. idahoensis* and instead find the *O. idahoensis* genome draft assembly is characterized by massive expansion of transposable elements.

Transposable elements are increasingly being studied for their ability to quickly generate novel genetic variation which may facilitate local adaptation [56]. Transposable element activity has been linked to rapid adaptive divergence in ants [57], several mammal species [58], *Anolis* lizards [59], and plants [60]. While the specific roles of transposable elements in the process of edaphic specialization remain to be elucidated within *Oreohelix,* our study suggests that LTRs, which compose more than half of the genome, have shaped three major genomic processes in *O. idahoensis*: genome size expansion, gene composition through retrotransposition of host genes, and ectopic recombination.

Similar to other large genome plant and animal species [60, 61], replication of LTRs and other transposable elements may be the primary driver of the large genome size of *O. idahoensis* compared to other land snail genera (e.g. *C. unifasciata* and *C. nemoralis*; Fig. 1) and other *Oreohelix* species (Figs. 1, 2 and 3). Our analysis of ancient and recent whole genome duplications lends support to this hypothesis as we detected no recent or ancient whole genome duplication specific to Oreohelicidae. Given the recent expansion of LTRs and other transposable elements in the *O. idahoensis* genome draft (Fig. 1) and large difference in repetitive k-mer content and genome size between genome skims of *O. idahoensis* and other *Oreohelix* species (Table 3), we conclude that the large genome size of *O. idahoensis* is a product of recent transposable element expansion.

The high proportion and recent activity of LTRs in the *O. idahoensis* genome suggests that these elements may be engines of genomic novelty which may contribute to the remarkable phenotypic variation present in *Oreohelix*. Retrotransposition of host genes by transposable elements has generated several retrocopies which have been incorporated as alternative splice variants of expressed host genes. Furthermore, we detected a high ratio of solo LTRs to intact elements (median family ratio: 6.33) which are typically produced through recombination between flanking LTR elements. Compared to plants which often have similar LTR content to *O. idahoensis*, the ratio of solo to intact elements in *O. idahoensis* genome draft is generally higher than most plant species [37] and may be indicative of increased genome wide ectopic recombination. Ectopic recombination between transposable elements can cause chromosomal translocations and inversions which can alter linkage relationships between genes and facilitate reproductive isolation between populations [62, 63]. When taken in context with our findings that the closely related non-limestone endemic *O. s. strigosa* contains much lower repetitive k-mer content than *O. idahoensis*, our results suggest that LTR mediated ectopic recombination could be a major mechanism underlying edaphic specialization in *Oreohelix*. However, it is also possible that stress associated with CaCO_3_ habitat specialization or secondary contact with neighboring species may facilitate decoupling of epigenetic control mechanisms on LTRs which may enable transposable element expansion [62]. Further research leveraging comparative genomic approaches on full genome assemblies of *O. idahoensis* and closely related smooth form non-limestone endemics are needed to test these hypotheses.

Gene family expansion analysis revealed several expanded gene families putatively related to stress/immunity (AIG1), DNA-packaging (histone H2A/H2B), and biomineralization (serine proteases and SERPINs) which are likely tied to the ecology and genomic architecture of *O. idahoensis.* The expansion of AIG1 genes may be related to the high frequency of parasites present in many *Oreohelix* populations [64] and thermal extremes that species experience in semi-arid montane environments [65]. Expansions of histone genes are often associated with greater epigenetic transcriptional control mechanisms [47]. The expansion of histone genes in *O. idahoensis* may permit a greater diversity of silencing mechanisms to regulate transposable element activity [66]. Serine proteases and SERPINs are involved in a variety of cellular processes (e.g. innate immunity) but are also two halves of a biomineralization system in which serine proteases promote crystal nucleation and SERPINs regulate the process [67]. These gene families are promising targets for future studies examining ecological specialization and genomic architecture in *Oreohelix*.

## Conclusions

We utilized genome skims and a hybrid de novo assembly approach to examine the genomic features associated with edaphic specialization to calcareous habitats in *Oreohelix* land snails. The limestone endemic *O. idahoensis* genome is the largest and most repetitive mollusc genome assembly published to-date and has substantial LTR content which has shaped genome size, ectopic recombination, and gene composition. The availability of this genome will facilitate studies into the molecular basis of heightened biomineralization in limestone environments and aids in the conservation genetics of this threatened group of land snails.

## Methods

### Sample collection and sequencing

Three species were sampled for this study: *O. idahoensis, O. jugalis*, and smooth *O. s. strigosa.* Specimens of *O. idahoensis* (10) and *O. jugalis* (2) were collected from a limestone outcrop and alluvial deposits at the limestone outcrop border, respectively, at Lucile, Idaho during three collection trips during the spring of 2017–2019. A single specimen of *O. s. strigosa* was collected from Barret Creek above the north shore of Lake Chelan, Washington in the fall of 2021. A single adult *O. idahoensis* specimen was selected for genome assembly and a portion of the abdominal region of the foot muscle (1.2 g) was removed and shipped on dry ice to the HudsonAlpha Institute for Biotechnology (Birmingham, AL) for total genomic DNA extraction and library preparation. DNA was extracted using a MagAttract HMW DNA Kit from Qiagen (PN- 67563) and a 10x linked read library was prepared using a Chromium v2 Genome Reagent kit (10X Genomics; PN-120258) with 2.5 ng of DNA as starting material. This same DNA extract was then shipped to the University of Idaho Institute for Bioinformatics and Evolutionary Studies Genomics Resource Core (IBEST-GRC) for continuous long-read (CLR) library prep for the PACBIO Sequel II system. The linked-read library was size selected for 450 bp insert size and sequenced on three Illumina HiSeq X Ten lanes producing 1.42 billion read pairs for a total yield of 425.8 Gb of linked reads. Fragment analyzer traces before and after CLR library prep confirmed the mean fragment length to be > 50 kb, the CLR library was sequenced using three Sequel II SMRT Cell 8 M’s producing 46 million reads totaling 324.5 Gb of unique molecular data.

Two separate RNA extractions were prepared from six pooled mantle-edge tissue and one whole body of *O. idahoensis* using TRIzol® Reagent (Life Technologies, Gaithersburg, USA) following manufacturer’s instructions. To check the quality of each RNA extract, we opted to assess RNA fragment length (Agilent systems M5310AA) over RNA integrity number (i.e. RIN) as there is no observable 28S peak for *Oreohelix* and many other gastropod species [68]. An equal proportion from each sample was combined for SMRT Bell sequencing on the PacBio Sequel I platform at the University of Washington PacBio Sequencing Services lab. Library preparation followed the Iso-Seq protocol with cDNA amplification of polyadenylated transcripts [69]. The two libraries were then pooled and sequenced using two Sequel I SMRT Cell 1 M v3’s. Consensus full-length non-chimeric (FLNC) sequences were called using SMRT Link v6.0.0 and output as fasta files for later analysis (https://www.pacb.com/support/softwaredownloads/).

To compare genome repetitiveness and genome size across *Oreohelix* species, we generated genome-skims for two *O. idahoensis*, two *O. jugalis*, and one *O. s. strigosa* sample. DNA was extracted from abdominal foot tissue for each sample using a Qiagen DNeasy blood and tissue kit and used input for library preparation using a NEBNext® Ultra DNA Library Prep Kit (PN- E7370L). All libraries were pooled and sequenced on a single 150 bp paired-end Novaseq S4 lane resulting in ~ 185 Gb of output.

### Genome feature estimation

As many assembly software packages require an estimate of genome size as a starting parameter for assembly (e.g. canu), we used a k-mer counting approach to estimate genome size using our Illumina linked read data. First, we removed the 10x molecule information encoded in the first 22 bp (16 bp barcode, 6 bp nmer-primer) of our Illumina paired-end linked-read data using longranger v.2.2.2 (github.com/10XGenomics/longranger). Scrubbed linked read pairs were then used to estimate genome size, heterozygosity, and repeat content through k-mer analysis. We counted the number of distinct canonical 31-mers using Jellyfish v.2.2.6 [70] and generated a histogram with a max coverage threshold of 1,000,000 for analysis using Genomescope v.2.0 [20]. We chose 31-mers for genome feature estimation using Genomescope v. 2.0 as higher k-mers are more suited to estimating genome size for highly repetitive genomes [20]. We also utilized ModEst on default parameters with our trimmed linked read-data which estimates genome size using mapping rates and coverage data of raw reads to genome assemblies to estimate genome size for *O. idahoensis* [21]*.* ModEst was not used for our genome skim data as there was insufficient coverage (< 5) to accurately estimate genome size with this approach when reads were aligned to individual draft assemblies constructed using the pipeline [21]. Instead, genome-skim data for all *Oreohelix* species were analyzed using RESPECT v.1.0.0 on default settings to estimate genome size and repeat content [36].

### Independent and hybrid assembly of linked and long reads

We utilized the 10x Genomics proprietary software Supernova v.2.1.1 [71] to generate a linked-read assembly using 2.1 billion reads, which is the maximum number of input reads that can be passed into Supernova at the time of this study (32 bit cap). All remaining parameters were kept at their default values. The resulting assembly was then output using the ‘pseudohap1’ option and a minimum contig length of 1 kb.

We opted to utilize the natively cluster capable genome assembly software canu v.1.9 [23] over other long-read assemblers given concerns that the high repeat content and large size of the *O. idahoensis* genome would likely lead to excessive run times. PacBio CLR long-reads were error corrected using canu v.1.9 [23] with the recommended settings for large and repetitive genomes: ‘corMhapFilterThreshold=0.0000000002 corMhapOpti”–=“--threshold 0–0 --num-hashes –2 --num-min-matche–3 --ordered-sketch-size 1–0 --ordered-k-mer-size–4 --min-olaplength 2–0 --repeat-idf-scal” 50” mhapBlockSize=500 ovlMerDistinct=0.975’. The unitgging step was performed using version 2.1.1 with the same assembly parameters as a new version of canu had been released since the initiation of the pipeline [72]. Initial polishing of the raw canu contigs using long reads and Racon v1.4.21 [73] resulted in a modest loss in BUSCO score so we opted to only polish using a single round of NextPolish v.1.3.1 [74] and the barcode-scrubbed linked reads created from longranger. All alignments required for polishing were done using minimap2 v.2.16 [75] unless otherwise stated. The polished canu assembly was then purged of duplicate regions and haplotig sequences using purge_dups on default settings [76].

We created a hybrid assembly following the previously published Supernova-DBG2OLC assembly pipeline [22]. Briefly, Supernova assembled contigs are aligned to uncorrected, compressed long-read contigs using DBG2OLC v1.0.0 [77] which then forms a backbone graph for assembly. This backbone graph is then output and available for polishing using the DBG2OLC consensus module or other user chosen polishing software. The Supernova-DBG2OLC backbone graph was generated using the following parameters in DBG2OLC: ‘k 17 AdaptiveTh 0.0001 K-merCovTh 2 MinOverlap 15 RemoveChimera 1 Contigs supernova_scaftigs.fa f CLR.fasta.’ We then polished the raw Supernova-DBG2OLC contigs using Racon for two rounds of long read polishing followed by one round of short read polishing using NextPolish.

Contigs were merged from both the canu and Supernova-DBG2OLC assemblies to produce a more contiguous assembly using quick-merge v.0.3 [78]. We chose to use the canu assembly as the query sequence and the Supernova-DBG2OLC as the reference to merge contigs as the canu assembly was more fragmented than the Supernova-DBG2OLC assembly. The two assemblies were merged by whole genome alignment using nucmer v.4.0.0rc1 [79] to identify overlaps (parameters: -l 100) and quick-merge using the following settings: ‘-hco 5 -c 1.5 -l 340000 -ml 10000.’ The merged assembly was then polished once with long reads and once using short reads using Racon and NextPolish, respectively. This polished assembly was then corrected for misassemblies using linked read barcode information by tigmint v.1.2.2 [80] on default settings except to set the max molecule length as 70,000 bp, which was slightly above the mean molecule length reported by Supernova (63,810 bp). The tigmint corrected assembly was then scaffolded using ARCS v.1.2.1 with default parameters [81]. Small scaffolds less than 5000 bp were then removed to facilitate faster repeat masking and gene annotation.

### Genome assembly quality evaluation

All assemblies were assessed for gene content completeness using the Benchmarking Universal Single Copy Ortholog v4.0.2 (BUSCO) metazoan gene set before and after polishing to ensure assemblies did not become overly polished [82]. The final merged and scaffolded assembly was screened for contaminants using conterminator v.1.0 [30] on the NCBI nonredundant nucleotide database (downloaded on October 26, 2021). Conterminator was run using default parameters except to only consider potential contaminations between *O. idahoensis* (NCBI:txid 2,584,915) and other taxa in the NCBI database. Identified contaminated regions were then blasted against the NCBI nonredundant nucleotide database to confirm contamination.

### Repeat identification and divergence

A de novo library of transposable elements and repeats was generated for *O. idahoensis* using the EDTA v1.9.9 pipeline on default settings [31]. EDTA is a combined structural and homology repeat detection pipeline that integrates several structural transposable element detection programs with homology searches using RepeatModeler 2.0 [34]. The EDTA transposable element library was then used for genome masking with RepeatMasker v4.1.1 [34] using rmblastn v2.2.28 (http://www.repeatmasker.org/RMBlast.html) as the search engine. A repeat landscape plot was then created using the transposable elements consensus alignments produced by RepeatMaskers and calculating the Kimura divergence from the consensus using the RepeatMasker script ‘calcdivergencefromalign.pl’ and the R-package ggplot2 [83].

To place the identified repeat content and divergence history of the *O. idahoensis* genome draft in context with other previously published mollusc genome assemblies, we generated individual EDTA repeat libraries for five other previously published mollusc genomes (*Cepaea nemorals, Candidula faciata, Achatina immaculata, Radix auricularia*, and *Euprymna scolopes*). These species were selected based on their close phylogenetic relatedness to *O. idahoensis* or for having a similar genome size (i.e. *Euprymna scolopes*). Each individual repeat library was used to mask and generate repeat landscape plots following the same procedure as the *O. idahoensis* genome draft.

LTR insertion times can be estimated by using a known mutation rate and comparing LTR divergence between sister pairs. We estimated the insertion times of intact LTR elements using a molluscan substitution rate of 1.645 × 10^− 9^ per site per year [84]. Insertion times of LTR elements were then placed in a phylogenetic context by comparing estimated divergence times of *Oreohelix* species from a previous study [16] with LTR insertion times estimated from the current study (Fig. 2).

We then investigated whether long terminal repeat transposons (LTR) were involved in reshaping structural variation in the *O. idahoensis* genome draft by estimating the proportion solo LTRs to intact LTR elements. LTRs are mobile transposable elements are composed of two flanking long terminal repeats and an internal sequence which enables replication through a copy-and-paste mechanism. Solo LTRs are formed by ectopic recombination between two LTR regions at non-homologous locations on the same or another chromosome [37]. The ratio of solo LTRs to intact LTRs can be used to estimate the level of ectopic recombination within the genome and deletion of LTR elements [37]. To measure the ratio of solo LTRs to intact LTRs in the genome assembly, we first used the ‘solo_finder.pl’ script from LTR_retriever [85] to identify solo LTR candidates. The ‘solo_finder.pl’ script reports solo LTR candidates have at least 80% coverage of an LTR element in the intact LTR library, possess with an alignment score greater than 300, and are at least 100 bp in length. Then, we filtered solo LTR candidates that were within 25 kb of a scaffold end as these may represent true intact elements that were not fully assembled. Finally, we calculated the ratio of intact vs solo LTR families using the ‘solo_intact_ratio.pl‘script from LTR_retriever.

### Genome annotation

We utilized information from Iso-Seq reads, protein homology, and ab initio predictions to annotate the *O. idahoensis* genome draft. The FLNC reads obtained from Iso-Seq sequencing were mapped to the soft-masked genome draft using minimap2, and transcript models were called using TAMA [86]. The mapped sequences were first collapsed with tama_collapse.py with the parameters ‘-s input.sam -f idaho_scaff_final.fa -p input.collapse -x no_cap -sjt 20 -lde 2 -a 100 -z 100’ for each library (mantle and whole body) and then merged together with the tama_merge.py script. The final set of assembled transcripts were then output as a fasta file use as transcript evidence for genome annotation.

We used MAKER (v.3.01.03) [87] to annotate the *O. idahoensis* genome in three rounds using transcript evidence from the TAMA processed Iso-Seq libraries, protein evidence from the *Cepaea nemoralis*, *Candidula unifasciata*, and *Achatina fulicula* genome assemblies, and the *O. idahoensis* EDTA repeat library to soft-mask transposable elements in gene models. In the first round, transcript and protein evidence were aligned to the genome draft using BLASTn and BLASTx [88] and refined for splice site alignment using Exonerate v2.4.0 to generate the initial set of gene models [89]. Given the large LTR content identified in the *O. idahoensis* genome draft (see below) and the positive correlation between LTR content and intron length across the tree of life [90, 91], we ran the first round of MAKER annotation with three values of the ‘split_hit’ parameter that controls max intron length (the default of 10,000 bp, 50,000 bp, and 100,000 bp). The transcriptome produced with a ‘split_hit’ value of 100,000 bp had the greatest number of complete BUSCO genes and was selected for further annotation refinement. MAKER employs annotation edit distance (AED) scores to assess the quality of generated gene models with respect to prior evidence (i.e. a lower AED score indicates greater agreement between gene model and protein/transcript evidence: 0 complete agreement, 1 no evidence). After the first round, 96.1% of the 22,384 genes had an AED score < 0.5 which indicates almost all gene models were well-supported by transcript or protein homology evidence. The second and third rounds of MAKER utilized the ab-initio predictors SNAP (version 2006-07-28) and Augustus v.3.3.3 [92] to generate new gene models. For each round, SNAP was trained on the prior rounds gene models that had AED scores lower than 0.25 and were of at least 50 amino acids in length. Augustus was retrained using the BUSCO metazoan gene set with the ‘-long’ parameter. The retrained Augustus models were then used to predict gene models during the second and third rounds of MAKER. For the final round of MAKER, we only retained gene models with AED scores < 0.5 resulted in 27,692 gene models. The resulting proteins from the gene models were then aligned against the NCBI nonredundant protein (downloaded on 26 October 2021), InterPro (accessed 26 October 2021) [29], and Uniprot databases (Swissprot and Trembl; accessed 26 October 2021). The longest isoform for each gene was then exported for protein phylogenetic reconstruction.

### Phylogenetic analysis and divergence time estimation

We placed the *O. idahoensis* genome draft in a phylogenetic context by first aligning single copy orthologs of several mollusc genome assemblies (*Pomacea canaliculata, Biomalpharia glabrata, Achatina fulica,* and *Candidula unifasciata,*) using Orthofinder v.2.4.0 with the ‘-M msa’ flag [38]. We then constructed an ultrametric time-calibrated tree using BEAST v.2.5.1 [39] with the following settings: WAG amino acid matrix, four gamma rate categories, estimate proportion of invariant sites and substitution rate, uncorrelated relaxed log-normal clock, birth-death model, runtime of 50,000,000 generations, burn-in of 10%, and setting tree height to be calibrated using the fossil date for the Caenogastropoda-Heterobranchia split (390 MYA offset log-normal distribution with a mean of 30) [40, 52]. Convergence was assessed visually using Tracer v.1.7.1 and effective sample size was confirmed to be greater than 200 for all parameters. We chose to omit the *C. nemoralis* genome assembly in our phylogenetic analysis due to the large number of genes in the *C. nemoralis* genome which may be a result of incomplete repeat masking [24, 25]. The inclusion of non-masked repetitive elements can affect downstream gene family expansion by altering estimates of ancestral gene family size between species [43].

### Gene family evolution

The orthogroup gene family counts generated by Orthofinder and the maximum clade credibility consensus BEAST ultrametic tree were used as input into CAFE v.3.03 [43] to examine gene family expansion across Gastropods. We estimated gene family expansion in CAFE by estimating a single birth-death parameter and a significance level of 0.01. Gene families identified as significantly expanding n *O. idahoensis* and containing GO annotations by InterProScan were then retained for GO enrichment analysis.

### Retrocopy identification

We scanned the genome for retrocopies produced through retrotransposition of host genes using Retroscan v.1.1 on that hard masked *O. idahoensis* genome assembly using default settings [49]. Retrocopies are partial or complete duplications of host genes but lack the introns of their parent sequence due to the mechanism of retrotransposition. Retrocopies inferred from retroscan correspond to four categories: (1) retrocopies that are entirely intact genes produced through retrotransposition which are labelled as ‘intact;’ (2) retrocopies that retain the open-reading-frame of the parent gene but recruit promoter and enhancer regions which make them functional are labelled ‘retrogenes;’ (3) retrocopies which have fused with other genes and are transcribed as introns or alternative splice variants of exons are labelled as ‘chimeric retrogenes;’ and (4) retrocopies with stop codons or no promoter regions are labelled as ‘pseudogenes.’ Expressed retrocopies were identified by blasting all retrocopies that did not contain a stop codon (retrogenes, chimeric retrogenes, and intact) to the TAMA assembled transcripts. Retrocopies were only considered expressed if they had an exact full-length match to one of the TAMA transcripts. All expressed retrocopies were then functionally annotated by InterProScan and those containing GO terms were retained for GO enrichment analysis.

### GO enrichment analysis

We utilized the R package clusterProfiler v3.14 to determine enriched GO terms in the expanded gene families and expressed retrocopies in the *O. idahoensis* genome [44]*.* First, full GO term ontologies were built using the ‘buildGOmap’ for foreground gene sets (e.g. expressed retrocopies) and the background gene set consisting of all genes (including retrocopies) within the *O. idahoensis* genome draft assembly. Then, GO enrichment analysis was performed using the ‘enricher’ function on default settings except to specify a Benjamini-Hochberg correction for *p*-value estimation (pvalueCutoff = 0.05, pAdjustMethod = “BH”).

### Detecting ancestral and recent whole genome duplication

To determine whether ancient whole-genome duplication has led to the large genome size of *O. idahoensis*, we generated Ks plots of whole paranome sequences using WGD v.1.0.1 [51]. Plots were examined visually for peaks in synonymous substitution values for paralogous genes in the *O. idahoensis* genome draft which may correspond to a genome duplication event. We estimated the timing of any inferred whole genome duplication events by dividing the estimated Ks peak value by a molluscan substitution rate of 1.645 × 10^− 9^ per site per year [84].

We also examined the possibility of a recent genome duplication by comparing allelic frequencies of heterozygous SNPs using nQuire v.1.0.0 [53]. In brief, nQuire estimates the best ploidy model (diploid, triploid, tetraploid) that fits the distribution of heterozygous SNPs mapped to the reference assembly by assuming diploids should have a frequency of 0.5/0.5, triploids 0.33/0.66, and tetraploids 0.25/0.75 and 0.5/0.5 [53]. We aligned the linked-reads scrubbed of 10x barcodes to the *O. idahoensis* genome draft assembly using minimap2 and then removed all alignments with a mapping quality less than 20. The bam file was then input into nQuire and ‘denoised’ of low frequency allele ratios following the recommendation of the authors [53]. The model of ploidy producing the lowest delta likelihood compared to the free Gaussian Mixture Model was then considered to be the ploidy of the *O. idahoensis* genome draft.

### Historical demography

Given the threatened conservation status of *O. idahoensis*, we also investigated historical demography of this species using PSMC [54]. We first generated input files for PSMC by using alignments of our barcode trimmed linked reads to our genome using minimap2 [75] and samtools mpileup v. 1.9 [93] to estimate heterozygous bases. We utilized a minimum mapping quality of 30 for samtools mpileup and required both pairs to align to the genome. PSMC was run on the input files for 20 generations and Ne was inferred across 74 time intervals (18 + 25*2 + 2 + 4) using default settings. PSMC results were scaled using the same molluscan substitution rate as previous analyses [84] and a generation time estimate taken from unpublished mark-recapture data (5.6 years; Tronstad, personal communication).

## Supplementary Information

**Additional file 1.**

**Additional file 2.**

**Additional file 3.**

**Additional file 4.**

**Additional file 5.**

**Additional file 6.**

**Additional file 7.**

## Data Availability

All sequence data used for this publication has been made available through NCBI BioProject accession number PRJNA727022. The genome assembly has been deposited in GenBank under JAMPIV000000000.1. The version used in the present study is 1.0.0. Annotation files, repeat libraries, and coding sequences are available on FigShare (10.6084/m9.figshare.c.6008425.v1).
