# Supplementary figures and images for: De novo genome assembly and genome skims reveal LTRs dominate the genome of a limestone endemic Mountainsnail (Oreohelix idahoensis)
Source: BMC Genomics. 2022 Dec 2;23:796. doi: 10.1186/s12864-022-09000-x (PMC9719178; doi:10.1186/s12864-022-09000-x)

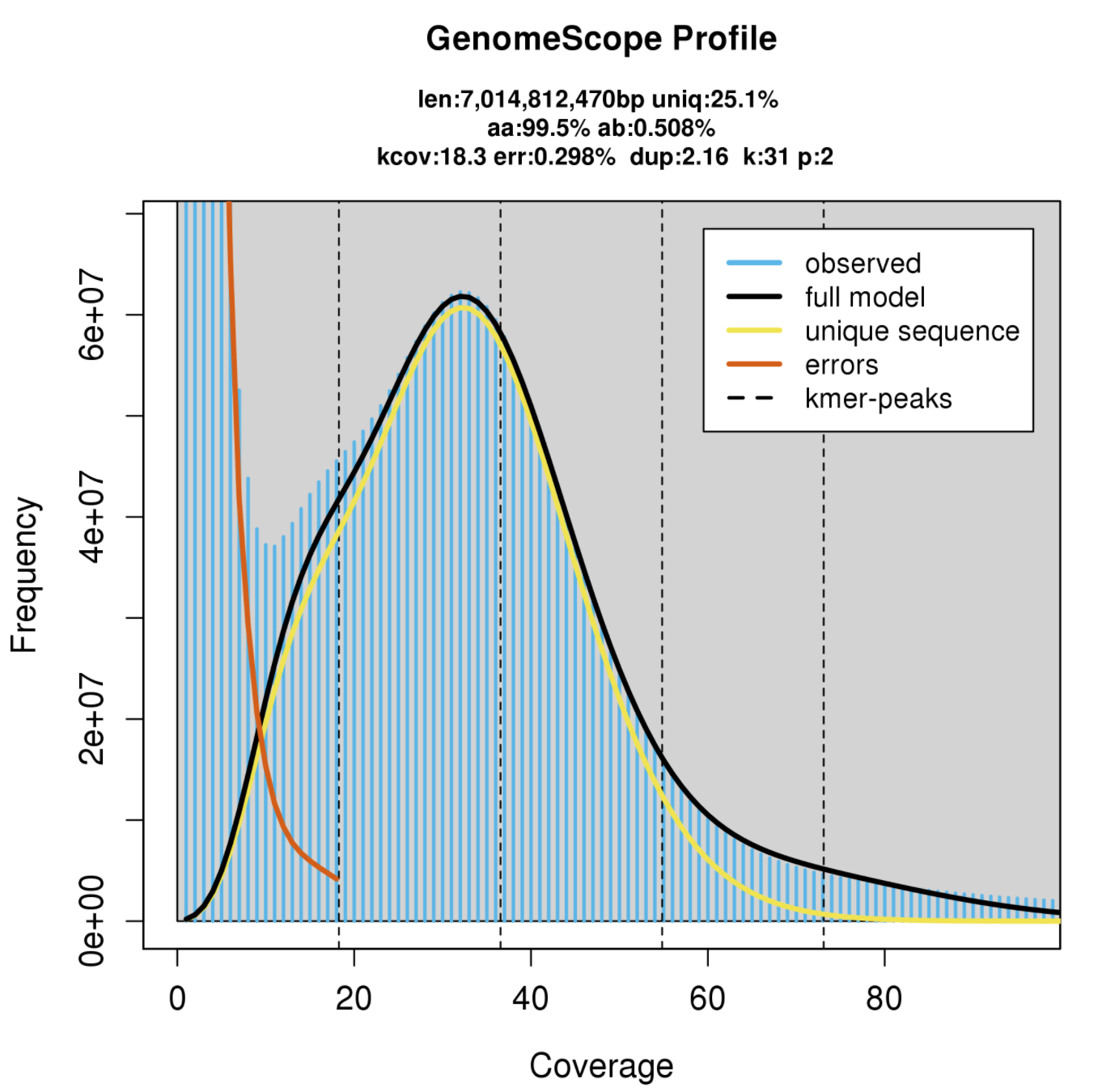

Supplement: Supplementary file 1 — Additional file 1. [file 12864_2022_9000_MOESM1_ESM.png]
